# Cerebellar rTMS disrupts predictive language processing

**DOI:** 10.1016/j.cub.2012.07.006

**Published:** 2012-09-25

**Authors:** Elise Lesage, Blaire E. Morgan, Andrew C. Olson, Antje S. Meyer, R. Chris Miall

**Affiliations:** 1 School of Psychology, University of Birmingham, B15 2TT, Birmingham UK; 2 Max Planck Institute for Psycholinguistics, Postbus 310, 6500 AH Nijmegen, The Netherlands

## Abstract

The human cerebellum plays an important role in language, amongst other cognitive and motor functions [1], but a unifying theoretical framework about cerebellar language function is lacking. In an established model of motor control, the cerebellum is seen as a predictive machine, making short-term estimations about the outcome of motor commands. This allows for flexible control, on-line correction, and coordination of movements [2]. The homogeneous cytoarchitecture of the cerebellar cortex suggests that similar computations occur throughout the structure, operating on different input signals and with different output targets [3]. Several authors have therefore argued that this ‘motor’ model may extend to cerebellar nonmotor functions [3–5], and that the cerebellum may support prediction in language processing [6]. However, this hypothesis has never been directly tested. Here, we used the ‘Visual World’ paradigm [7], where on-line processing of spoken sentence content can be assessed by recording the latencies of listeners' eye movements towards objects mentioned. Repetitive transcranial magnetic stimulation (rTMS) was used to disrupt function in the right cerebellum, a region implicated in language [8]. After cerebellar rTMS, listeners showed delayed eye fixations to target objects predicted by sentence content, while there was no effect on eye fixations in sentences without predictable content. The prediction deficit was absent in two control groups. Our findings support the hypothesis that computational operations performed by the cerebellum may support prediction during both motor control and language processing.

## Main Text

**Figure 1:**
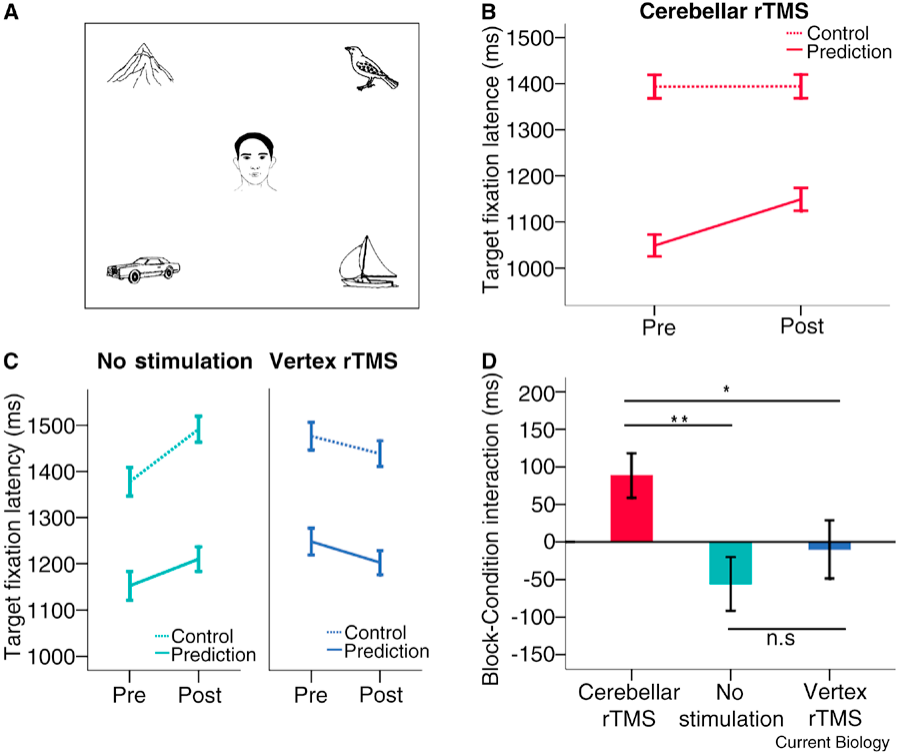
Stimulus example and eye movement analysis results. (A) Example of a visual scene. In the Prediction condition (e.g. “The man will sail the boat”), the direct object of the sentence (the boat) can be predicted from the verb “sail” because it is the only object in the array plausibly related to that action. In the Control condition (e.g. “The man will watch the boat”), such prediction is not possible. (B) Target fixation latencies before and after rTMS to the right lateral cerebellum. Participants were slower to fixate the target in the Prediction condition (solid red), while fixation latency in the Control condition (dashed red) was unaffected. (C) Target fixation latencies in the No Stimulation condition (left) and the Vertex rTMS condition (right). There was no interaction between Block and Condition in either group. (D) Block-by-Condition interactions for the three groups. The hypothesized positive interaction is only evident in the cerebellar group (red), and is significantly different from the two control groups (green and blue), which do not differ from each other. (B–D) Error bars in all panels denote ±1 standard error of the mean. ^∗^: p < 0.05; ^∗∗^: p < 0.01; n.s.: no significant difference.

We recorded the eye movements of 65 participants while they listened to pre-recorded sentences and looked at static displays depicting the agent and the direct object (the target) of the sentence, as well as three distracter objects which were not mentioned in the sentence (Figure 1A). In half the trials, the target object could be predicted from the verb (Prediction condition), in the other half such prediction was not possible (Control condition). We measured the time it took listeners to look at the target object (the target fixation latency) from the onset of the verb. Earlier studies using similar materials have shown that listeners are considerably faster to fixate the target object in the Prediction condition than in the Control condition [7]. In 22 participants, rTMS was applied to the right cerebellum between two blocks of Visual World task trials (for details on the materials, design, stimulus randomization, and TMS protocol see the Supplemental Information available on-line with this issue). If the cerebellum is engaged in on-line linguistic prediction, the disruption of this mechanism should slow down target fixation when prediction is possible (Prediction trials). Conversely, when target prediction is not possible (Control trials) the disruption should not affect target fixation latency.

As hypothesized, participants were slower to fixate the target following cerebellar rTMS in the Prediction condition, but were not slowed in the Control condition (rTMS-by-Condition interaction: F(1,21) = 8.848, p = 0.007, repeated-measures ANOVA; Figure 1B). That is, disrupting function in the right cerebellum selectively impaired the prediction aspect of sentence processing in this task; other language processes were spared. This effect cannot be explained by changes in eye movement kinematics, which were not altered by rTMS (see Supplemental Information).

To ensure that the slower fixation in the Prediction condition was not due to effects of fatigue, familiarity with the task, or an effect of rTMS not specific to the cerebellum, we performed two control experiments. One group of participants (n = 21) received rTMS over a control site, the vertex, and another group (n = 22) received no TMS stimulation at all. Repeated-measures ANOVAs showed that the Block-by-Condition interaction was absent in both the Vertex Stimulation control condition (F(1,20) = 0.064, p = 0.802) and the No Stimulation control condition (F(1,21) = 2.461, p = 0.132). An analysis using data from all three groups revealed a significant three-way interaction (F(2,62) = 4.548, p = 0.014). Planned comparisons between the groups were carried out using t-tests and demonstrate that the Block-by-Condition interaction in the cerebellar rTMS group differed significantly from that in both the No Stimulation control group (t(42) = 3.111, p = 0.003) and the Vertex rTMS control group (t(41) = 2.021, p = 0.050), while the interaction did not differ significantly between the two control groups (t(41) = 0.875, p = 0.387; Figure 1C,D and Supplemental Information). We can therefore attribute the impaired performance in the Prediction condition to disruption of neural operations by rTMS over the right cerebellum. There is no reason to believe that rTMS effects on neighboring structures, including the right occipital lobe, could be responsible for this selective deficit in predictive processing.

Finally, to ensure the fixation latency effects observed were not due to an inability to perform the task following cerebellar rTMS, we also analyzed error rates before and after rTMS, and between the two conditions after cerebellar rTMS. If the participants were unable to identify the spoken words, or if the information flow between language centers involved in sentence comprehension and oculomotor centers involved in object fixation was disrupted, this would be reflected in the fixation behavior of participants. Neither of the error measures differed significantly from the first to the second block or between conditions after rTMS (see Supplemental Information). Hence, cerebellar rTMS resulted in an equally accurate, but delayed target fixation, consistent with the loss of the temporal advantage conferred by a short-term prediction.

It is thought the cerebellum contributes to fast and flexible motor control by predicting the sensory consequences of movements on a fine timescale, and that these predictions are available before visual or proprioceptive feedback from the executed actions [2,4]. This cerebellar ‘forward model’ prediction, based on an efferent copy of motor commands, allows for rapid error detection and correction, for motor coordination and motor planning [2,4,6]. Like motor control, language comprehension is highly time sensitive, and listeners must process the spoken input on-line, at a rate set by the speaker. While they cannot anticipate with certainty what will be said, they can often predict future sentence content based on shared linguistic and world knowledge [7]. A predictive process similar to forward modeling in motor control [4] could therefore contribute to the speed and efficiency of language processing [9].

We cannot yet say how these predictions are made; they might directly predict semantic content or instead they might predict internalized speech production that could indirectly support comprehension [9]. There is evidence that the right lateral cerebellum (lobule HVII/Crus I) is part of the verbal working memory and language system [1,10] (see also Supplemental Information). In addition, the right lateral cerebellum is connected with cortical language and higher cognitive areas such as Broca's area and the dorsolateral prefrontal cortex [1,5,8]. So we speculate that input to the right cerebellum from connected language structures, possibly Broca's area, would provide an ‘efferent copy’ of internalized speech, from which the lateral cerebellum would predict future speech output. These predictions then would feed back to frontal cortical language areas to facilitate processing, in parallel to cortico-cortical inputs. Several aspects of the predictive role of the cerebellum in language processing should be further investigated. There is evidence that some aspects of language processing are embodied, and interact with motor control processes. We cannot yet say whether the effect we report here also shows sensitivity to action-related verbs. In our study, the predictions were based on the verb meanings. Future research should also investigate whether other types of prediction based, for instance, on syntactic or pragmatic information also implicate the cerebellum.

In summary, we have shown that language processing is delayed in a predictive language task when right cerebellar function is disrupted. Our data suggest that the cerebellar theory of predictive motor control can be extended to the nonmotor cerebellum. This adds support to the notion that similar computations are performed across the structure, with different inputs and with different output targets.

## Supplemental Information

Document S1. Figure S1 and Supplemental Experimental Procedures
