# Supplementary material for: Cerebellar rTMS disrupts predictive language processing
Source: Curr Biol. 2012 Sep 25;22(18):R794–5. doi: 10.1016/j.cub.2012.07.006 (PMC3459089; doi:10.1016/j.cub.2012.07.006)
Supplement: Document S1. Figure S1 and Supplemental Experimental Procedures [file mmc1.pdf]

Supplemental information: Cerebellar rTMS disrupts predictive language processing

Lesage, E., Morgan, B.E., Olson, A.C., Meyer, A.S., and Miall, R.C.

INVENTORY

Supplemental Data

Figure S1: Error rates for the cerebellar rTMS group between blocks, and between conditions after cerebellar rTMS.

Supplemental Experimental Procedures

1. We provide a description of the materials, task, and stimulation protocol used in this study.
2. We present Supplemental Analysis and Results sections, detailing the main statistical analysis, as well as additional analyses.
3. A final section provides background information on cerebellar involvement in language.

SUPPLEMENTAL FIGURE

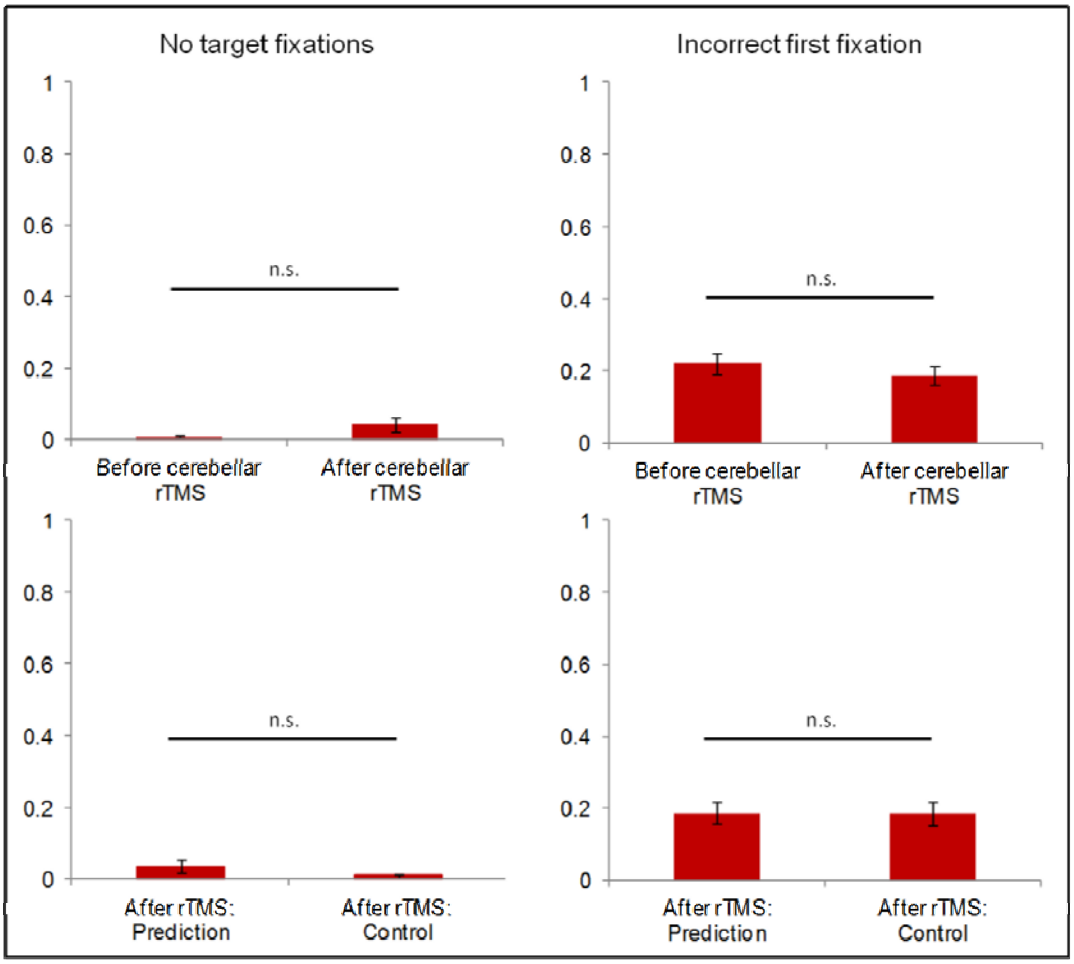

Figure S1: Error analysis.

Average proportion of trials where the target object was not fixated at all (left column) and the average proportion of trials where a non-target object was fixated prior to the target object (right column). The upper row is collapsed across both conditions; the lower row separates the Prediction condition from the Control condition after cerebellar rTMS. On both measures, there was no change in participants' performance following cerebellar rTMS, nor did they perform better or worse in the Prediction condition following rTMS. These data indicate that the participants' accuracy in identifying the target words was not affected by the stimulation.

SUPPLEMENTAL EXPERIMENTAL PROCEDURES

1. Supplemental Methods

All methods and procedures were approved by the local ethics committee, and written informed consent was obtained for each participant.

Participants

All participants were right-handed native English speakers recruited from the University of Birmingham student population, and had no contra-indications for TMS, as assessed by a standard screening form (http://prism.bham.ac.uk/downloads/TMS_screening_form.pdf). The 65 participants (mean age = 20.5 years, 20 male) received course credit or financial compensation for their time.

Task details

At the beginning of each Visual World trial, four objects appeared, one in each corner of the screen. Participants were instructed to look at all objects, think about what they were and where they were on the screen. After 3000ms, the face of the agent (a man, woman, boy or girl) appeared in the middle of the screen, and as soon as the participant fixated the face of the agent, a pre-recorded sentence was played over headphones. Participants were instructed to look at what was mentioned in the sentence.

Materials

Auditory stimuli were 64 spoken sentences, constituting 32 items. Sentences were spoken by an English native speaker with a neutral accent. They were digitally recorded as .wav files and played over standard headphones. Of the 64 sentences, 28 were taken from Altmann and Kamide [1]. The remaining 36 sentences were newly created.

Each item consisted of one sentence in the Prediction condition and one sentence in the Control condition. The only difference between Prediction and Control sentences was the

verb, which could refer to only one of the objects in the display (Prediction condition), or to any of the four objects in the display (Control condition). The display was identical in both conditions. The two verbs were matched for frequency of occurrence using the CELEX data base [S1].

Randomization

The 32 items were divided across two blocks of 16 items. The Prediction and Control variant of each item appeared in the same block so that the eye movements before and after rTMS stimulation could be compared within participants and within items. The order of the blocks was counterbalanced between participants. Within each block, the presentation of the items was pseudorandom, such that the Prediction and the Control version of the sentence pair were never presented adjacently.

Eye tracking

Eye movements were recorded using a desk-mounted Eyelink 1000 apparatus sampling the right eye at 1000 Hz. Each block was preceded by a 12 point eye tracker calibration. Drift correction was performed between trials. Each Visual World block lasted under 10 minutes.

Stimulation protocol

TMS stimulation was delivered using a Magstim Rapid apparatus and lasted 10 minutes. There was less than two minutes between the end of the stimulation and the start of the next experimental block. In the two participant groups where TMS pulses were delivered, the stimulator intensity was set at a fixed level of 55% of maximum stimulator output (MSO). We opted to use a fixed intensity rather than an intensity proportional to motor threshold, as primary motor cortical excitability is uncorrelated to the excitability of tissue elsewhere in the brain [S2, S3].

Cerebellar rTMS

The stimulation site was 1cm down and 3cm to the right from the inion. This is a site frequently used to stimulate lateral areas of the cerebellum [S4] and data from our lab using a large number of structural MRI scans has shown that this location on the scalp is closest to cerebellar tissue. The target site was stimulated at a fixed 55% of maximum stimulator output intensity with a double-cone coil for 10 minutes at 1Hz (600 pulses). Low-frequency rTMS has been shown to produce LTD-like after-effects in the targeted tissue for a period lasting up to the length of the stimulation [S5]. Prior to the stimulation, one single pulse was delivered in order to familiarize the participant with the sensation, and to allow them the opportunity to withdraw their consent prior to the rTMS stimulation.

Vertex rTMS control

The control stimulation site was taken as the Cz (the vertex), measured as the mid-point between the two external auditory canals in the transverse plane and the mid-point between inion and nasion in the saggital plane. The control site was stimulated at a fixed 55% of maximum stimulator output intensity with a flat figure-of-eight coil for 10 minutes at 1Hz (600 pulses). Prior to the stimulation, one single pulse was delivered in order to familiarize the participant with the sensation, and to allow them the opportunity to withdraw their consent prior to the rTMS stimulation.

No Stimulation control

Participants in the No Stimulation group were given instructions and explanations as if they were going to receive cerebellar rTMS. After the first Visual World block, they were informed that no TMS pulses would be delivered. However, the cerebellar stimulation site was determined and the double-cone coil was placed over this site for 10 minutes, without activation of the coil.

2. Supplemental Analysis

The dependent variable used was the target fixation latency; the time from the onset of the verb to the onset of the first fixation of the target object (the object of the sentence). We analysed fixations from 400ms after verb onset onwards, as it would take the participant at least 400 ms to understand the verb and initiate a saccade [S6, S7]. The reported latencies are measured from verb onset. Trials where no target fixation was made before 3500ms after verb onset were discarded from the latency analysis. Repeated measures ANOVAs and mixed effect modelling were performed on the fixation latencies (see below). In addition to the analyses of fixation latencies, we also compared error rates and several eye-movement kinematic parameters (average saccade velocity, peak saccade velocity, and saccade duration) before and after cerebellar rTMS using paired t-tests.

Repeated measures ANOVA

Repeated measures ANOVAs with fixed factors Block (levels Pre- and Post TMS) and Condition (levels Prediction and Control) and with participants as a random variable were conducted for each of the three groups of participants using SPSS software.

Mixed Model Analysis

It has been proposed that a linear mixed model approach is a more appropriate analysis strategy for this type of psycholinguistic data [S8]. The specific items used in this study represent a subsection of a larger population of possible experimental items, and could be treated as a random factor in addition to the random factor Participant. A repeated-measures ANOVA does not take these issues into account.

Therefore, in addition to the analyses of variance we performed linear mixed effect modelling on the fixation latency data using the lme4 package in R. This allowed us to enter both Participant and Item simultaneously as random effects [S9, see also S8], which is not possible in analyses of variance. Condition and Block were entered as fixed effects. For this

analysis, Block was treated as a time-varying variable, with values 0 for the first block, and 1 for the second block. A model selection strategy using likelihood ratio tests was used to find the best fitting model with the least parameters [S8]. When the most appropriate random effects structure was selected, models with different combinations of fixed effects were compared.

Error rate comparison

To ensure the fixation latency effects observed were not due to an inability to perform the task following rTMS, we also analysed error rates before and after cerebellar rTMS, and between the two conditions after cerebellar rTMS. If the participants were unable to identify the spoken words, or if the information flow between language centres involved in sentence comprehension and oculomotor centres involved in object fixation was disrupted, this would be reflected in the fixation behaviour of participants. "No-fixation" trials were defined as trials in which the correct target object was not fixated within 3500 ms; these trials have been excluded from the analysis of fixation latencies. We also determined the proportion of "error" trials: those in which the target was not the first object fixated, but where valid target fixation latency was measured within the trial duration; these trials have been included in the latency analyses.

Supplemental Results

Repeated-measures ANOVA

While the Cerebellar rTMS group showed a significant interaction between Block and Condition ($F(1,21) = 8.848$, $p = 0.007$; see main article), this interaction was absent in both the Vertex Stimulation control condition (Block-by-Condition interaction: $F(1,20) = 0.064$, $p = 0.802$; Repeated-measures ANOVA) and the No Stimulation control condition (Block-by-Condition interaction: $F(1,21) = 2.461$, $p = 0.132$; Repeated-measures ANOVA). An analysis using data from all three groups revealed a significant three-way interaction (See main article).

Eye movement kinematics

None of the measured eye movement variables showed any difference after cerebellar rTMS. Paired t-tests for average saccade velocity (t(21) = 1.26, p = 0.222), peak saccade velocity (t(21) = -0.04, p = 0.972), and saccade duration (t(21) = -1.64, p = 0.116) did not reach significance, indicating that there were no TMS-induced effects on eye movement kinematics, and ruling these out as a possible cause of post-TMS changes in target fixation latencies.

Mixed Model Analyses

The additional linear mixed model analyses revealed the same pattern of results as the ANOVA approach reported in the main article and above. In the Cerebellar Stimulation group, model comparison favoured a model with a Block-by-Condition interaction against the simpler model without an interaction (χ^2 = 4.100, p = 0.043). In the Vertex Stimulation control group (χ^2 = 0.013, p = 0.908) and in the No Stimulation control group (χ^2 = 1.044, p = 0.307), the model with the interaction was not preferred, and therefore the simple, no interaction model should be assumed. The preferred random effects structure included Item as a random effect on the intercept, and Participant as a random effect on both intercept and slope.

Error rate analysis

Pair-wise t-tests comparing the number of errors before and after cerebellar rTMS revealed no significant effects, with a trend towards fewer errors after rTMS, possibly due to greater familiarity with the task (Paired t-test: t(21) = 1.916, p = 0.069; see Figure S1, right column). Following rTMS, the error rates did not differ between the two predictive conditions (Paired t-test: t(21) = -0.075, p = 0.941). When comparing trials where no fixations to the target were made, no significant differences arose between blocks before and after cerebellar rTMS (Paired t-test: t(21) = -1.867, p = 0.076), or between the two predictive conditions following

cerebellar rTMS (Paired t-test: $t(21) = -1.336$, $p = 0.196$; Figure S1, left column). These results demonstrate that participants were equally able to identify the target words and make corresponding eye movements. This further indicates that the observed rTMS-by-Condition interaction was due to a deficit in prediction, which resulted in slower fixation of the target on Prediction trials.

3. Supplemental information: Involvement of the cerebellum in language processing

A considerable body of evidence shows cerebellar involvement in language. Below we briefly review neuropsychological evidence from cerebellar lesions and dyslexia studies, as well as evidence for connectivity between cortical and cerebellar language areas, and neuroimaging studies in healthy control participants.

Patients with cerebellar lesions may present with problems with lexical access and syntax [S10], and speech production deficits [S11]. These deficits are have been interpreted as a failure of a cortico-cerebellar loop, which includes frontal and cerebellar language areas [S12, 8]. Such accounts are consistent with the idea that cortico-cerebellar loops support higher-level processes [S13].

Dyslexia has been linked to cerebellar deficits [S14, S15]. Eckert et al. [S16] analysed structural MRI scans of dyslexic children and controls, and found that the volume of the pars triangularis (Broca's area) and the right anterior cerebellum distinguished dyslexics from non-dyslexics. As in the lesion literature, this evidence is consistent with a higher-order cortico-cerebellar language system.

Cortical language areas, particularly Broca's area, have been found to be connected to the cerebellum. Patients with right cerebellar lesions show selective hypoperfusion in Broca's area [8]. A recent fMRI study of functional connectivity found strong bidirectional connectivity between the right cerebellum and both left inferior frontal gyrus and left middle temporal gyrus [S17]. Evidence from resting state functional connectivity studies demonstrates

connections between frontal, parietal and temporal areas and the lateral cerebellum [S18, S19].

In whole-brain neuroimaging studies of language in healthy participants, right cerebellar activity is often found. PET and fMRI studies of word generation [S20, S21], word stem completion [S22, S23], verbal fluency [S24], and semantic judgment [S25, S26, S27, S28, S29] elicit activity in the right cerebellum, along with cortical language areas such as the left inferior frontal gyrus and the left middle temporal gyrus. In a review of 100 fMRI studies on language [S30] semantic processing was found to activate left cortical language regions and the right cerebellum. Recently, Fedorenko et al., [S31] validated an fMRI language localizer, designed to detect language areas in individual subjects. The right cerebellum was amongst the regions which reliably (on an individual subject basis) responded to sentences versus non-word strings, whether there was a motor task component or not, and whether the stimuli were presented visually or aurally. While many of these studies do include motor aspects, these are controlled for in the contrasts used (i.e. there is an equal amount of covert speech or button presses in each condition). In a quantitative meta-analysis of various tasks that elicit cerebellar activation, language tasks were located to the right lateral cerebellum [S32]. These right lateral regions, distinct from medial regions controlling eye movements, were the target of our study.

To the best of our knowledge, no fMRI or EEG studies have investigated predictive language processing in the cerebellum. In fMRI studies of semantic prediction and priming, the cerebellum is rarely imaged or analysed. If cerebellar activations are found, these tend not to be discussed [S30, S33]. EEG signals from the cerebellum are very difficult to obtain due to its anatomical location. As far as we know, no EEG language studies have investigated the cerebellum. However, an ERP study by Dien et al. [S34] has localized a "meaningfulness" component to the right cerebellum, consistent with a cerebellar role in lexical access. Unfortunately, the exact source location is difficult to pinpoint using standard source localisation algorithms, as Dien and colleagues [S34] point out.

The present literature supports for a role for the right cerebellum in semantic processing, which is independent of modality in input (acoustic or visual stimuli) and output (overt speech, covert speech, button presses). Cerebellar lesions tend not to result in aphasic symptoms, but instead cause relatively mild impairments. No imaging studies have investigated a cerebellar role in linguistic prediction specifically, but the present literature is consistent with a cerebellar role in feedforward linguistic prediction, as has been proposed by Pickering and Garrod [10]. This hypothesis was therefore tested in our study.

SUPPLEMENTAL REFERENCES

S1. Baayen, R.H., Piepenbrock, R., and Gulikers, L. (1995). The CELEX lexical database (CD-ROM). University of Pennsylvania, Philadelphia, PA: Linguistic Data Consortium.

S2. Stewart, L.M., Walsh, V., and Rothwell, J.C. (2001). Motor and phosphene thresholds: a transcranial magnetic stimulation correlation study. Neuropsychologia *39*, 415-441.

S3. Boroojerdi, B., Meister, I.G., Foltys H., Sparing, R, Cohen, L.G., and Topper, R. (2002). Visual and motor cortex excitability: a transcranial magnetic stimulation study. Clin. Neurophysiol. *113*, 1501-1504.

S4. Theoret, H., Haque, J., and Pascual-Leone, A. (2001). Increased variability of paced finger tapping accuracy following repetitive magnetic stimulation of the cerebellum in humans. Neurosci. Lett. *306*, 29-32.

S5. Chen, R., Classen, J., Gerloff, C., Celnik, P., Wasserman, E.M., Hallet, M., and Cohen, L.G. (1997). Depression of motor cortex excitability by low-frequency transcranial magnetic stimulation. Neurology *48*, 1398-1403.

S6. Marlsen-Wilson, W., and Tyler, L.K. (1980). The temporal structure of spoken language understanding. Cognition *8*, 1-71.

S7. Matin, E., Shao, K., and Boff, K. (1993). Saccadic overhead: information processing with and without saccades. Percept. Psychophys. *53*, 372-380.

S8. Baayen, R.H., Davidson, D.J., and Bates, D.M. (2008). Mixed-effects modelling with crossed random effects for subjects and items. J. Mem. Lang. *59*, 390-412.

S9. Bates, D.M. (2005). Fitting linear mixed models in R. R News *5*, 27-30.

S10. Fabbro, F., Moretti,R., and Bava, A. (2000). Language impairments in patients with cerebellar lesions. J. Neurolinguistics *13*, 173-188.

S11. Silveri, M.C., Leggio, M.G., and Molinari, M. (1994). The cerebellum contributes to linguistic production - a case of agrammatic speech following a right cerebellar lesion. Neurology *44*, 2047-2050.

S12. Mariën, P., Saerens, J., Nanhoe, R., Moens, E., Nagels, G., Pickut, B.A., Dierckx, R.A., and De Deyn, P.P. (1996). Cerebellar induced aphasia: case report of cerebellar induced prefrontal aphasic language phenomena supported by SPECT findings. J. Neurol. Sci. *144*, 34-43.

S13. Kelly, R. M., and Strick, P. L. (2003). Cerebellar loops with motor cortex and prefrontal cortex of a nonhuman primate. J. Neurosci. *23*, 8432-8444.

S14. Nicholson, R.I., and Fawcett, A.J. (2011). Dyslexia, dysgraphia, procedural learning and the cerebellum. Cortex *47*, 117-127.

S15. Ivry, R.B., and Justus, T.C. (2001). A neural instantiation of the motor theory of speech perception. Trends Neurosci. *24*, 513-515.

S16. Eckert, M.A., Leonard, C.M., Richards, T.L., Aylward, E.H., Thomson, J., and Berninger, V.W. (2003). Anatomical correlates of dyslexia: frontal and cerebellar findings. Brain *126*, 482-494.

S17. Booth, J.R., Wood, L., Lu, D., Houl, J.C., and Bitan, T. (2007). The role of the basal ganglia and cerebellum in language processing. Brain Res. *1133*, 136-144.

S18. O'Reilly, J. X., Beckmann, C. F., Tomassini, V., Ramnani, N., and Johansen-Berg, H. (2010). Distinct and Overlapping Functional Zones in the Cerebellum Defined by Resting State Functional Connectivity. Cereb. Cortex *20*, 953-965.

S19. Buckner, R.L., Krienen, F.M., Castellanos, A., Diaz, J.C., and Yeo, T.B.T. (2011). The organization of the human cerebellum estimated by intrinsic functional connectivity. J. Physiol. *106*, 2322-2345.

S20. Lurito, J.T. Kareken, D.A. Lowe, M.J., Chen, S.H.A., and Mathews, V.P. (2000). Comparison of rhyming and word generation with FMRI. Hum. Brain Mapp. *10*, 99-106.

S21. Frings, M., Dimitrova, A., Schorn, C.F., Elles, H-G, Hein-Kropp, C., Gizewski, E.R., Diener, H.C., and Timmann, D. (2009). Cerebellar involvement in verb generation: An fMRI study. Neurosci. Lett. *406*, 19-23.

S22. Ojemann, J.G., Buckner, R.L., Akbudak, E., Snyder, A.Z., Ollinger, J.M., McKinstry, R.C., Rosen, B.R., Petersen, S.E., Raichle, M.E., and Conturo, T.E. (1998). Functional MRI studies of word-stem completion: Reliability across laboratories and comparison to blood flow imaging with PET. Hum. Brain Mapp. *6*, 203-125.

S23. Buckner, R.L., Koutstaal, W., Schacter, D.L., and Rosen, B.R. (2000). Functional MRI evidence for frontal and inferior temporal cortex in amodal components of priming. Brain *123*, 620-640.

S24. Schlosser, R., Hutchinson, M., Joseffer, S., Rusinek, H., Saarimaki, A., Stevenson, J., Dewey, S.L., and Brodie, J.D. (1998). Functional magnetic resonance imaging of human brain activity in a verbal fluency task. J. Neurol. Neurosurg. Psychiatry *64*, 492-498.

S25. Seger, C.A., Desmond, J.E., Glover, G.H., and Gabrieli, J.D.E. (2000). Functional magnetic resonance imaging evidence for right-hemisphere involvement in processing unusual semantic relationships. Neuropsychology *14*, 361-369.

S26. McDermott, K.B., Petersen, S.E., Watson, J.M., and Ojemann, J.G. (2003). A procedure for identifying regions preferentially activated by attention to semantic and phonological relations using functional magnetic resonance imaging. Neuropsychologia *41*, 293-303.

S27. Xiang, H.D., Lin, C.Y., Ma, X.H., Zhang, Z.Q., Bower, J.M., Weng, X.C., and Gao, J.H. (2003). Involvement of the cerebellum in semantic discrimination: An fMRI study. Hum. Brain Mapp. *18*, 208-214.

S28. Tieleman, A., Seurinck, R., Deblaere, K., Vandemaele, P., Vingerhoets, G., and Achtena, E. (2005). Stimulus pacing affects the activation of the medial temporal lobe during a semantic classification task: An fMRI study. NeuroImage *26*, 565-572.

S29. Binder, J.R., Frost, J.A., Hammeke, T.A., Cox, R.W., Rao, S.M., and Prieto, T. (1997). Human brain language areas identified by functional magnetic resonance imaging, J. Neurosci. *17*, 353-362.

S30. Price, C.J. (2010). The anatomy of language: a review of 100 fMRI studies published in 2009. Proc. Natl. Acad. Sci. USA *1191*, 62-88.

S31. Fedorenko, E., Hsieh, P-J, Nieto-Castañón, A., Whitfield-Gabrieli, S., and Kanwisher, N. (2010). New Method for fMRI Investigations of Language: Defining ROIs Functionally in Individual Subjects. J. Neurophysiol. *104*, 1177–1194.

S32. Stoodley, C. J., and Schmahmann, J. D. (2009). Functional topography in the human cerebellum: A meta-analysis of neuroimaging studies. NeuroImage *44*, 489-501.

S33. O'hare, A.J., Dien, J., Waterson, L.D., and Savage, C.R. (2008). Activation of the posterior cingulate by semantic priming: A co-registered ERP/fMRI study. Brain Res. *1189*, 97-114.

S34. Dien, J., Frishkoff, G.A., Cerbone, A., and Tucker, D.M. (2003). Parametric analysis of event-related potentials in semantic comprehension: evidence for parallel brain mechanisms. Cogn. Brain Res. *15*, 137-153.
